# hAECs and their exosomes improve cardiac function after acute myocardial infarction in rats

**DOI:** 10.18632/aging.203066

**Published:** 2021-05-24

**Authors:** Yi-Qing Zhang, Lu Hong, Yu-Feng Jiang, Sheng-Da Hu, Nan-Nan Zhang, Lang-Biao Xu, Hong-Xia Li, Gui-Dong Xu, Ya-Feng Zhou, Kang-Yun Sun

**Affiliations:** 1 Department of Cardiology, The Affiliated Suzhou Hospital of Nanjing Medical University, Suzhou Municipal Hospital, Gusu School, Nanjing Medical University, Suzhou, Jiangsu Province, P.R. China; 2 Department of Cardiology, DuShu Lake Hospital Affiliated to Soochow University, Suzhou, Jiangsu Province, P.R. China

**Keywords:** human amniotic epithelial cells, exosome, acute myocardial infarction, rat

## Abstract

Background: Human amniotic epithelial cells (hAECs) are seed cells used to treat acute myocardial infarction (AMI), but their mechanism remains unclear.

Methods: We cultured hAECs and extracted exosomes from culture supernatants. Next, we established a stable AMI model in rats and treated them with hAECs, exosomes, or PBS. We assess cardiac function after treatment by echocardiography. Additionally, heart tissues were collected and analyzed by Masson’s trichrome staining. We conducted the tube formation and apoptosis assays to explore the potential mechanisms.

Results: Cardiac function was improved, and tissue fibrosis was decreased following implantation of hAECs and their exosomes. Echocardiography showed that the EF and FS were lower in the control group than in the hAEC and exosome groups, and that the LVEDD and LVESD were higher in the control group (P<0.05). Masson’s trichrome staining showed that the fibrotic area was larger in the control group. Tube formation was more efficient in the hAEC and exosome groups (P<0.0001). Additionally, the apoptosis rates of myocardial cells in the hAEC and exosome groups were significantly decreased (P<0.0001).

Conclusions: hAECs and their exosomes improved the cardiac function of rats after AMI by promoting angiogenesis and reducing the apoptosis of cardiac myocytes.

## INTRODUCTION

Mortality from cardiovascular diseases has been increasing recently, accounting for approximately one-third of all deaths in the population, and has posed a hefty burden to the social economy, according to the American Heart Association [1]. Therapies for myocardial infarction have gradually developed in recent years. Thrombolysis, coronary artery bypass grafting (CABG) and percutaneous coronary intervention (PCI) could rescue myocardial cells that are close to death; however, heart failure occurs inevitably [2]. Thus, strategies to repair dying myocardial cells and improve prognosis are needed. Scientists have focused increasingly on cell therapy, and many cell types have been demonstrated to improve cardiac function after acute myocardial infarction (AMI), such as bone marrow-derived stromal cells, embryonic stem cells, mesenchymal stem cells and induced pluripotent stem cells [3]. However, the application of these cells remains limited because the cell survival rate is low *in vivo* and the cells can become tumorigenic or cause inflammatory problems [4, 5]. Accumulating evidence has shown that stem cells function through autocrine, paracrine and other ways and play a role in infarcted myocardium repair. Scientists have gradually abandoned the concept of stem cells by proliferation differentiation into cardiomyocytes as a protection [6].

Exosomes are important factors secreted by stem cells. Because of their advantages of low immune exclusion and easy access, stem cell-derived exosomes have attracted increasing attention as a new noncellular therapy [7]. Exosomes are disk-like vesicles of varying sizes, with a diameter of approximately 30-150 nm. They play an important role in the intercellular communication process through the lncRNAs, siRNAs, miRNAs and mRNAs contained within them, as well as proteins, DNA, lipids and other substances [8]. The functions of exosomes are gradually being revealed. Studies have reported that exosomes secreted by stem cells have similar biological functions as maternal stem cells [9, 10].

Human amniotic epithelial cells (hAECs) are human amniotic stem cells that can be isolated from human placental tissue and show low immunogenicity, low tumorigenicity and anti-inflammatory properties [11]. Studies by Yi-Sun Song and Yu-Ying Wang have shown that hAECs improve the cardiac function after myocardial infarction in rats by differentiating into cardiac myocytes, but the mechanism remains unclear [12, 13]. Research on exosomes has been increasing since 2010, when Lai et al. isolated exosomes and used them to treat mice with AMI and found that exosomes could significantly reduce the myocardial infarction area and might play a protective role in promoting angiogenesis and inhibiting apoptosis [14]. Lee WH et al. demonstrated that exosomes secreted by embryonic stem cells and human induced pluripotent stem cells contain small RNAs (miRs) and long noncoding RNAs (lncRNAs) that can promote angiogenesis and significantly improve cardiac function after myocardial infarction in nude mice [15].

Exosome and hAECs have good prospects in treating acute myocardial infarction, and the few related studies have shown controversial results. Therefore, we further explored the effect of hAECs and exosomes secreted by hAECs on acute myocardial infarction.

## MATERIALS AND METHODS

### Cell culture

Human primary amniotic epithelial cells were provided by Icell (Shanghai, China; http://www.icellbioscience.com/index), human umbilical vein endothelial cells (HUVECs) and myocardial cells (H9C2 cells) were purchased from the Stem Cell Bank (Chinese Academy of Science; http://www.cellbank.org.cn/). All the cells were cultured in complete medium comprising 89% Dulbecco’s modified Eagle’s medium (DMEM; Invitrogen) supplemented with high glucose, 10% fetal bovine serum (FBS; GIBCO) and 1% penicillin/streptomycin (GIBCO). We selected P4-P6 hAECs for our next experiment, and the conditioned medium was collected to extract exosomes.

### EdU labeling and hAEC staining

The P4-P6 hAECs were labeled with EdU, an analogue of thymidine nucleoside (T). In the cell proliferation phase, EdU is substituted for T infiltration during the replication of the DNA molecule. Based on the specificity of the fluorescent dyes in the EdU and using the Apollo^®^ system, cell proliferation could be detected using the EdU tag. We added EdU according to the manufacturer’s instructions of the EdU kit (Ribobio, https://www.ribobio.com/).

### Isolation and identification of exosomes

The conditioned medium of P4-P6 hAECs was collected in a 50-ml centrifuge tube and centrifuged at 2000 g for 30 minutes to remove dead cells and other cell fragments. Next, the supernatant was transferred to another aseptic centrifuge tube, followed by the addition of 0.5 volumes of exosome isolation reagent (Thermo Fisher Scientific) at 4° C. After 24 hours, the mixture was passed through a 0.22-μm cell filter, and then we placed the mixture in a centrifuge for 1 hour at 10000 g, discarded the supernatant, leaving the exosomes on the wall of the centrifuge tube. Phosphate-buffered saline (PBS) was used to resuspend the exosomes and store them at -80° C for the next step. The protein concentration of the extracted component was detected using the bicinchoninic acid (BCA) protein assay (Solarbio, China). CD63(BD) and CD81(BD) were identified by flow cytometry (Accuri C6 flow cytometer; BD). We observed the lipid bilayer vesicles using electron microscopy (H-7650; Hitachi) and conducted nanoparticle tracking analysis using the ZETASIZER Nano series-Nano-ZS.

### PKH-26 labeling and exosome staining

The exosomes were labeled with PKH-26 (Sigma), a fluorescent dye widely used to label exosomes, according to the manufacturer’s instructions. Next, the labeled exosomes were stored at -80° C for implantation into rats with acute myocardial infarction.

### Induction of myocardial infarction, cells and exosome transplantation

We purchased SD rats (male, 270–320 g) from Soochow University Experimental Animal Center and conducted animal experiments according to the Guide for the Care and Use of Laboratory Animals published by the U.S. National Institutes of Health (NIH publication No. 85–23, revised 1996). Twenty-four rats were randomly divided into three groups: a control group treated with PBS, a group treated with hAECs and a third group treated with exosomes. We generated the acute myocardial infarction model as previously reported [16] with a slight modification. Briefly, the rats were anesthetized with 4% chloral hydrate. First, we performed tracheotomy and connected a breathing machine (instrument plant of Chengdu, China), and then electrocardiography (ECG) followed by thoracotomy was conducted. After exposing the heart well, we ligated it to the left anterior descending (LAD) coronary artery. We estimated the modeling by the changes in the ventricular wall activity and color and ST segment changes of ECG. We transplanted 150 μl of PBS, 1.5*106 hAECs suspended with 150 μl of PBS or 300 μg of exosomes suspended with 150 μl of PBS into 3 sites around the infarcted heart tissue 30 minutes after myocardial infarction. Finally, the thoracic incision was closed carefully. After awakening, the rats were placed back into the cage for intensive monitoring.

### Measurement of cardiac function and myocardial infarction size

Echocardiography was performed at baseline and 7, 14 and 28 days after MI surgery by a blinded researcher well trained in the procedure. We used a 250-Hz transducer (SONOS 7500; Philips Medical Systems) and measured the left ventricular end-diastolic diameters (LVEDDs), left ventricular end-systolic diameters (LVESDs) and ejection fraction (EF) and fraction shortening (FS) in 3 consecutive cardiac cycles.

The rats were euthanized 28 days after myocardial infarction, and their heart tissue for Masson’s trichrome staining (Sigma) was removed to measure the size of the infarcted tissue. Briefly, the harvested heart tissues were trimmed carefully and immersed in 4% formaldehyde for 2 days at 4° C and then were transferred to 10%, 20% and 30% sucrose solution for gradual dehydration at 4° C, over a 24-hour period. Next, we used OCT (Sakura) component to embed the tissues and then froze them in dry ice. We then prepared frozen sections of heart tissue perpendicular to the left anterior descending coronary artery with a slice thickness of approximately 6 mm. We stained the sections according to standard procedures, and then the area of infarction was measured using ImageJ pro software (http://www.mediacy.com).

### Fluorescence immunohistochemistry

To detect the distribution and activity of transplanted cells and exosomes, the rats were euthanized 7 days after myocardial infarction and the heart tissue was removed for fluorescence immunohistochemistry. The specific treatment of the heart tissue was the same as above. The frozen sections from the hAEC group were stained with EdU, followed by observation under a fluorescence microscope (Olympus). Because PKH-26 is fluorescent, the frozen sections from the exosome group could be observed directly under a fluorescence microscope.

### Tube formation assay

Before the tube formation assay, we co-cultured hAECs (cell group) and exosomes (exosome group) using the same generation of HUVECs and then set up a control group, HUVECs without treatment. After coculturing for 48 hours, the prepared HUVECs were used in the tube formation assay. First, we added 50 μl of growth factor-reduced Matrigel (BD Biosciences) to 96-well plates and then stored it in an incubator for 30 minutes at 37° C. At the same time, we prepared HUVECs at the appropriate concentration and then the prepared HUVECs were seeded at a density of 50,000 cells per well of a 96-well plate. After the Matrigel solidified, we incubated the plate at 37° C. After 8 hours, we observed the images of tube formation using an inverted microscope (Olympus) and counted all the interconnecting tubes in each group using ImageJ pro software.

### Apoptosis detection of myocardial cells

We co-cultured hAECs (cell group) and exosomes (exosome group) with the same generation of H9C2 cells for 48 hours and set up a control group, H9C2 cells without treatment. Next, we replaced the medium with sugar-free medium and placed the H9C2 cells of each group into the oxygen-free environment for 5 hours at 37° C. We detected the apoptosis of H9C2 cells by flow cytometry. Annexin V-FITC/PI dyes distinguish dead cells, apoptosis cells and live cells according to different staining patterns. We stained H9C2 cells according to the manufacturer’s instructions of the Annexin V-FITC/PI kit (BD) and used flow cytometry (Beckman FC 500) to analyze the rate of apoptosis cells.

### Statistical analysis

All the data in the experiment were presented as means ± standard deviation, and t test was used for comparison analysis between the experimental and control groups. P<0.05 indicated statistical significance between the groups. One-way ANOVA was used for comparison among the groups. SPSS 15.0 and GraphPad Prism 8.0 statistical software packages were used to analyze the data.

## RESULTS

### hAECs were cultured, and the exosomes were extracted

**Figure 1:**
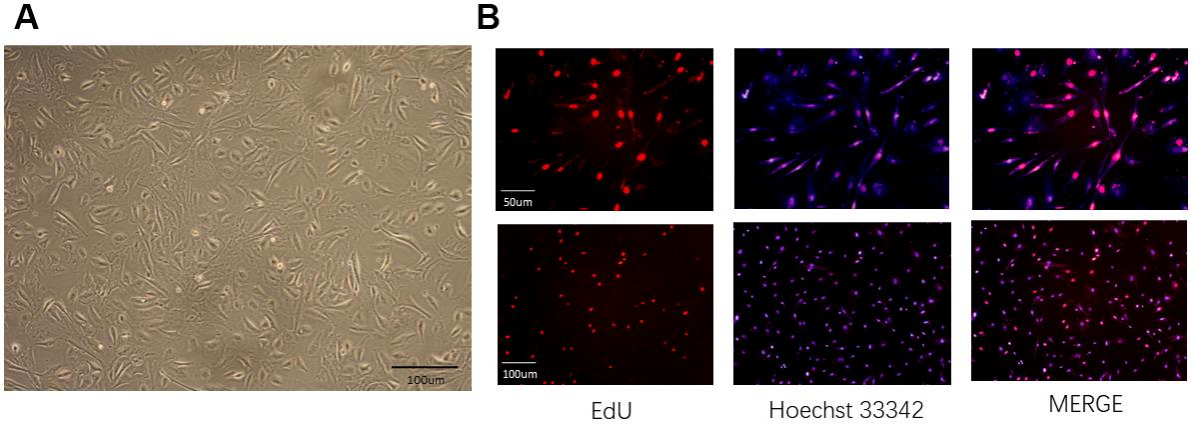
**Characterization and labeling of human amniotic epithelial cells (hAECs).** (**A**) The morphology of hAECs was observed under a microscope. (**B**) Immunofluorescence of hAECs labeled with EdU *in vitro*.

**Figure 2:**
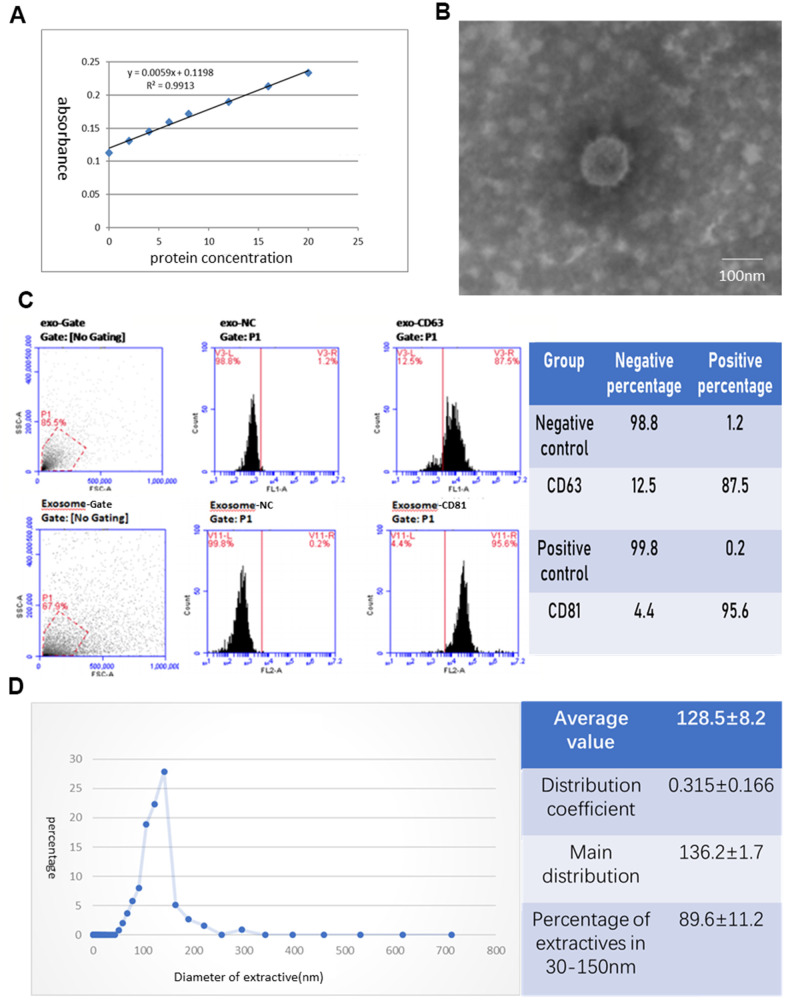
**Identification of exosomes deprived from hAECs.** (**A**) Protein concentration curve from the BCA test. (**B**) The morphology of exosomes was observed under an electron microscope. (**C**) The phenotype of exosomes for CD63 and CD81 was identified by flow cytometry. (**D**) The diameter distribution of exosomes was measured by nanoparticle tracking analysis.

hAECs were polygonal and showed adherent growth ability (Figure 1A). The cell proliferation rate slowed down significantly after P8, and individual cells grew larger and flattened. Therefore, P4-P6 cells were selected for all experiments. We labeled the hAECs to be implanted *in vivo* by EdU to identify them by *in vitro* immunofluorescence (Figure 1B). We extracted exosomes from 300 μg of the supernatants from each T75 cell culture bottle according to the manufacturer’s instructions of the exosome extraction kit (Figure 2A) and confirmed their identity in subsequent assays. Electron microscopy showed that the exosomes were oval and round, with variable sizes of lipid-containing cystic vesicles (Figure 2B). The results of surface marker antigen staining of CD63 and CD81 showed that the positive labeling rates were 86.2±1.4% and 94.9±1.1%, respectively (Figure 2C), and the average value (128.5±8.2 nm) and main distribution (136.2±1.7 nm) of the extracted material were within the range of exosomes (Figure 2D). These results prove that exosomes were extracted, provided a reliable guarantee for subsequent analysis.

### The cardiac function is improved by implanting hAECs and their exosomes in a rat acute myocardial infarction model

**Figure 3:**
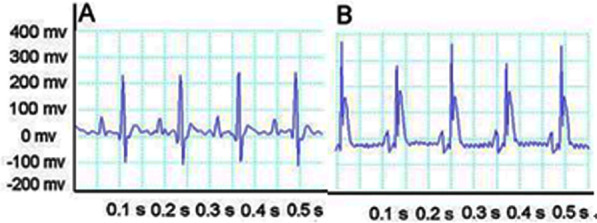
**ECG shows typical dynamic changes.** (**A**) ECG before ligating the coronary artery. (**B**) After ligation, the ST of ECG is elevated.

**Figure 4:**
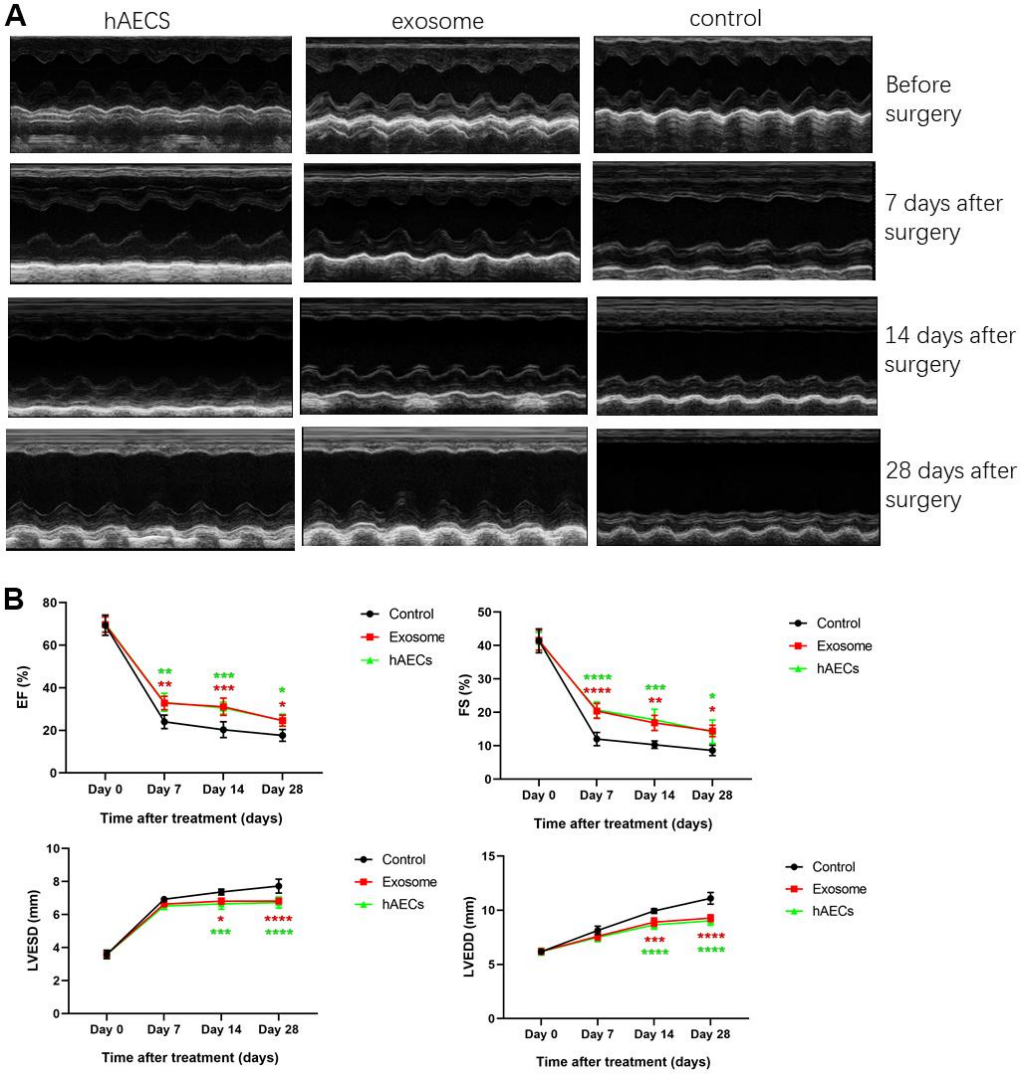
**Echocardiography shows that cardiac function is improved by implanting hAECs and their exosomes in a rat acute myocardial infarction model.** (**A**) Representative M-mode echocardiography of SD rats before and after MI in the hAEC treatment group, exosome group and control group. (**B**) The line charts shows the analysis of the left ventricular ejection fraction (EF), left ventricular fractional shortening (FS), left ventricular end-diastolic diameter (LVEDD) and left ventricular end-systolic diameter (LVESD) in SD rats 7, 14 and 28 days after MI, respectively. The data are expressed as the means ± SD, n=8 per group, *P<0.05.

**Figure 5:**
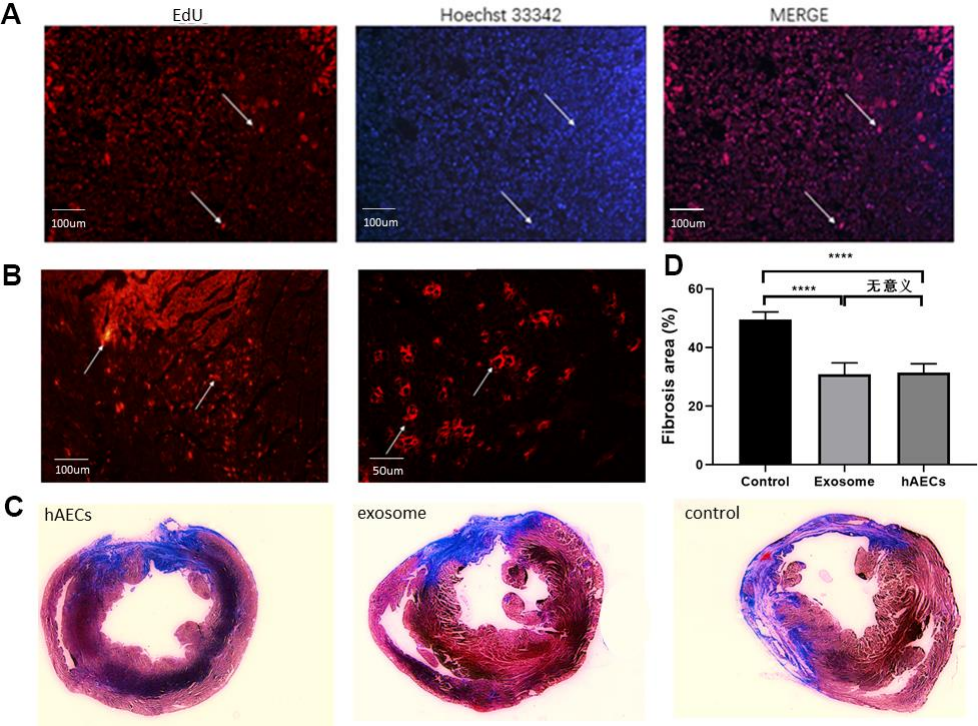
**hAECs and exosomes implanted in the heart decrease the fibrosis area.** (**A**) Immunofluorescence of hAECs *in vivo* 7 days after MI are shown. (**B**) Immunofluorescence of exosomes *in vivo* 7 days after MI are shown. (**C**) Representative images of four consecutive myocardial slices stained with Masson’s trichrome in the hAEC, exosome and control groups 28 days after MI are shown. (**D**) The bar graph shows quantitative analysis of the LV fibrosis area. The data are shown as the means ± SD, n=4, *P<0.05.

We established the stable model of acute myocardial infarction in rats. After ligating the LAD coronary artery, ECG showed typical dynamic changes such as changes in ST. Compared with the ST of ECG before coronary artery ligation (Figure 3A), the ST of ECG was elevated approximately 5 minutes after ligating the LAD (Figure 3B). Next, we implanted hAECs and exosomes from the hAEC culture supernatant into the myocardium of acute myocardial infarction rats and set up a control group to avoid the influence of other factors on the results. We then supervised the alteration of cardiac function via echocardiography (Figure 4). The typical graph of each group in the M-mode is shown in Figure 4A, and the ultrasound data were measured at baseline and 7, 14 and 28 days after MI surgery. The postoperative EF and FS were lower in the control group than those in the therapy group (P<0.0001). The LVEDD and LVESD measured at 14 and 28 days postoperatively were significantly higher in the control group than those in the therapy group (P<0.05; Figure 4B). Fluorescence immunohistochemistry confirmed that the hAECs and exosomes were scattered in the myocardial tissue at 7 days after surgery (Figure 5A). Masson’s trichrome staining revealed that the area of tissue fibrosis in the control group was larger at 28 days after surgery, and the difference between groups was significant (P<0.0001; Figure 5B). The infarction area is displayed in Figure 5C. Taken together, the results showed that the hAECs and exosomes secreted by hAECs could improve cardiac function after AMI and decrease the infarction area.

### The protective mechanism is explored *in vitro*

**Figure 6:**
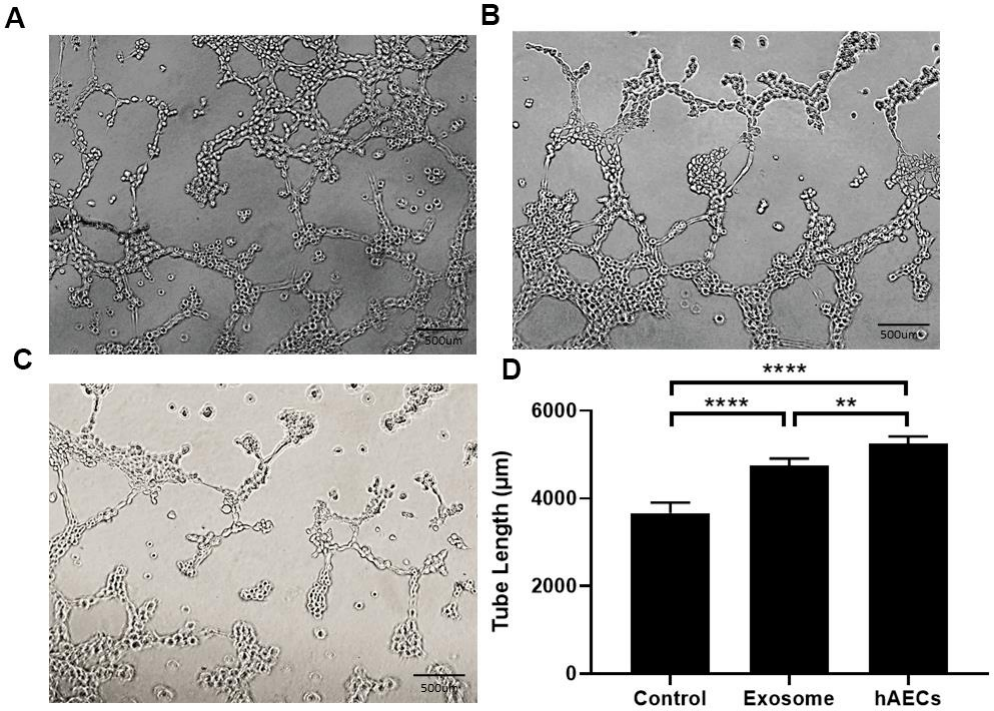
**Lumen formation and quantitative analysis of the lumen length.** Both hAECs and exosomes promote lumen formation. (**A**) Treatment with hAECs. (**B**) Treatment with exosomes. (**C**) Control group. (**D**) The bar graph shows quantitative analysis of the tube length. The data are shown as the means ± SD, n=4, *P<0.05.

Finally, the therapeutic mechanisms were explored. We conducted the tube formation assay to assess the potential mechanism of HUVECs and found that both hAECs and exosomes promoted lumen formation ability. In the control group, the HUVECs rarely formed tubes (Figure 6). The lumen lengths measured using image J pro Plus 6.0 were as follows: cell group (Figure 6A): 5251±166.09 μm; exosome group (Figure 6B): 4755.8±157.52 μm; control group (Figure 6C): 3669±236.03 μm. The difference between the experimental and control groups was statistically significant (P<0.05; Figure 6D).

**Figure 7:**
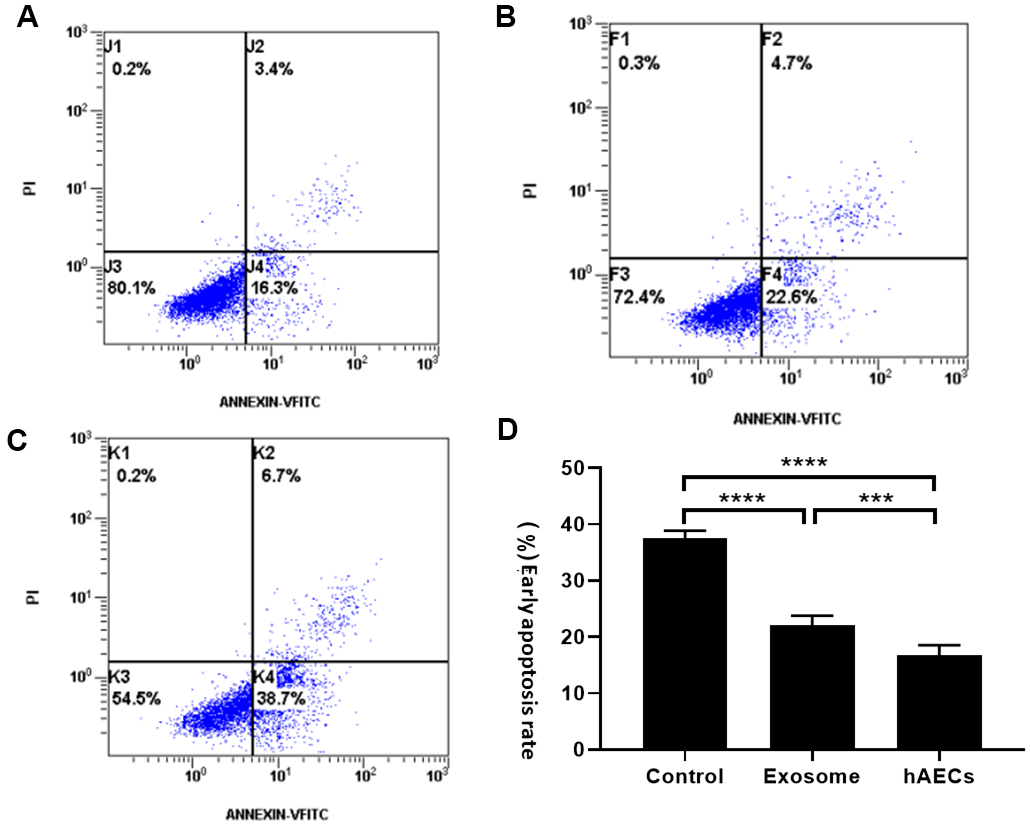
**The apoptosis rate of cardiomyocytes was measured by flow cytometry.** The results showed that both hAECs and exosomes decrease apoptosis. (**A**) Treatment with hAECs. (**B**) Treatment with exosomes. (**C**) Control group. (**D**) The bar graph shows quantitative analysis of early apoptosis. The data are shown as the means ± SD, n=4, *P<0.05.

Apoptosis plays a major role in AMI. In this experiment, we mainly explored the protection of hAECs and exosomes. The results of cardiomyocyte apoptosis were similar to those of tube formation. Following co-culture with hAECs and exosomes, the apoptosis rate of H9C2 decreased significantly compared with that of the control group. In an oxygen-free environment for 5 hours, the apoptosis rate of each group was as follows: cell group (Figure 7A): 16.76±1.77%; exosome group (Figure 7B): 22.04±1.71%; control group (Figure 7C): 37.46±1.4%. The difference between each group was statistically significant (P<0.05; Figure 7D).

## DISCUSSION

We showed that both hAECs and exosomes improved the cardiac function of rats after acute myocardial infarction and that cardiac function and the myocardial infarction area were similar between treatment with cells or exosomes. Thus, hAECs play a protective role in the treatment of acute myocardial infarction by secreting exosomes. Although many studies have reported that exosomes improve cardiac function, the specific underlying mechanisms remain unclear [17–19]. We conducted the apoptosis detection of myocardial cells and tube formation assay to explore the potential mechanisms *in vitro* and found that promoting angiogenesis and inhibiting cardiac apoptosis were the protective mechanisms.

hAECs, with strong plasticity, multidirectional differentiation potential, low immunogenicity, low tumorigenic and anti-inflammatory characteristics, have been considered a potential candidate to treat myocardial infarction [11]. Recent studies have demonstrated that hAECs improve cardiac function after myocardial infarction in rats by differentiating into cardiomyocytes [12, 13]; however, the mechanism requires further exploration. Exosome-related studies have gradually increased in recent years, and scientists have reached a consensus that the protection of stem cells is not only related to paracrine and autocrine factors but also influenced by extracellular vesicles, such as exosomes, which are secreted by stem cells [19–21]. Exosomes, whose components include microRNAs, lncRNAs, and siRNAs, are considered a novel therapy in regenerative medicine [22, 23]. Echocardiography showed that the EF and FS in the therapy group were significantly higher than those in the control group 7, 14 and 28 days after surgery, indicating that the treatment was effective. However, the meaningful decrease in the EF and FS at 28 days compared with 7 days was likely due to the single treatment, ventricular remodeling and expansion of the cardiac cavity, which reduced cardiac function. The LVEDD and LVESD measured 14 and 28 days postoperatively were obviously reduced in the therapy group compared with that in the control group, suggesting that the implanted hAECs and exosomes secreted by hAECs after myocardial infarction can inhibit ventricular remodeling and improve heart function in rats. By contrast, no conspicuous difference was observed in 7 days; ventricular remodeling was not evident, and the measurements indicated no prominent changes. After 28 days postoperatively, although the LVEDD and LVESD in the therapy group were significantly improved, ventricular remodeling still occurred; thus, the cardiac function decreased over time, a finding that was similar to the previous research results of Wang et al. [13]. These results offer new evidence that cardiac function is improved by implanting hAECs and their exosomes in the rat model of acute myocardial infarction.

To clarify the phenomenon demonstrated *in vivo* and explore the potential mechanisms, we performed relevant *in vitro* studies. We simulated myocardial infarction *in vitro* by building an H9C2 ischemic hypoxemia model [24], in which the tube formation assay for angiogenesis [25] was also conducted. We observed that hAECs and exosomes decreased apoptosis and increased angiogenesis *in vitro*. Additionally, treatment with hAECs was more effective than with exosomes. However, the *in vivo* results differed, showing that treatment with hAECs and exosomes had similar efficacies. *In vivo*, we implanted hAECs and exosomes at different doses, and the *in vitro* intervention procedure was the same. The concentration of implanted exosomes was 300 μg and that of implanted hAECs was 1.5*106 *in vivo*. However, 1.5*106 hAECs could extract only 100 μg of exosomes, likely explaining the divergent outcomes. We considered that exosomes play a positive role in acute myocardial infarction, in addition to other bioactive factors, such as vascular endothelial growth factor, fibroblast growth factor and circulating microRNAs, which could improve cardiac function [26–30]. We deemed that the role of hAECs was not only to produce exosomes in a protective function but also to cooperate with various biological activity factors. The mystery of exosomes needs more basic experiments to be gradually uncovered. Additionally, the stability, safety and bioavailability of exosomes require further study [31, 32].

We demonstrated that hAECs improved cardiac function after acute myocardial infarction, and the mechanisms of protection include inhibiting apoptosis and promoting angiogenesis. Exosomes, as an important component of stem cell secretion, with many protective substances, high availability, and low immunogenicity, are a potential candidate for therapy [15, 20, 22, 31–33]. Many studies have investigated exosomes. Zhao et al. found that rat bone marrow mesenchymal-derived exosomes can reduce inflammatory infiltration and fibrosis in the myocardial infarction area and promote the survival of rat embryonic myocardial cells (H9C2) under the oxidative stress of H2O2. The specific mechanism is that these exosomes can upregulate mir-24 and mir-29, downregulate mir-130 and mir-34, and further affect Ras, PI3K/Akt, mammalian target protein of rapamycin and other pathways to play a protective role [33]. Arslan et al. demonstrated that exosomes derived from mesenchymal stem cells could enhance myocardial vitality, prevent adverse remodeling after myocardial ischemia/reperfusion injury, and activate the PI3K/Akt pathway by increasing the ATP level to reduce oxidative stress [34]. Therefore, exosomes have great potential in regenerative medicine [35].

Because of experimental limitations, our experiment only proves that hAECs and their exosomes can improve cardiac function after myocardial infarction in rats by promoting angiogenesis and reducing myocardial cell apoptosis; the specific underlying pathway and other potential protective mechanisms have not been explored. In the future, the source of exosomes and mechanism of cell protection and differentiation mediated by exosomes in cardiovascular diseases will be explored.
